# Anticoagulants impact on innate immune responses and bacterial survival in whole blood models of *Neisseria meningitidis* infection

**DOI:** 10.1038/s41598-018-28583-8

**Published:** 2018-07-05

**Authors:** Lea Strobel, Kay O. Johswich

**Affiliations:** Institute for Hygiene and Microbiology, University of Wuerzburg, Wuerzburg, Germany

## Abstract

*Neisseria meningitidis* (meningococcus) causes invasive diseases such as meningitis or septicaemia. *Ex vivo* infection of human whole blood is a valuable tool to study meningococcal virulence factors and the host innate immune responses. In order to consider effects of cellular mediators, the coagulation cascade must be inhibited to avoid clotting. There is considerable variation in the anticoagulants used among studies of *N*. *meningitidis* whole blood infections, featuring citrate, heparin or derivatives of hirudin, a polypeptide from leech saliva. Here, we compare the influence of these three different anticoagulants, and additionally Mg/EGTA, on host innate immune responses as well as on viability of *N*. *meningitidis* strains isolated from healthy carriers and disease cases, reflecting different sequence types and capsule phenotypes. We found that the anticoagulants significantly impact on cellular responses and, strain-dependently, also on bacterial survival. Hirudin does not inhibit complement and is therefore superior over the other anticoagulants; indeed hirudin-plasma most closely reflects the characteristics of serum during *N*. *meningitidis* infection. We further demonstrate the impact of heparin on complement activation on *N*. *meningitidis* and its consequences on meningococcal survival in immune sera, which appears to be independent of the heparin binding antigens Opc and NHBA.

## Introduction

*N*. *meningitidis* is a normal constituent of the normal bacterial flora of the upper respiratory tract mucosa in 10–20 percent of the human population^1^. However, several hyper-virulent lineages of these Gram-negative bacteria are feared for their ability to spread from their mucosal niche into the bloodstream where they survive and divide, giving rise to life-threatening invasive meningococcal disease (IMD) with clinical pictures of meningitis and fulminant meningococcal sepsis^2^. Particularly the latter one is characterized by extremely rapid progression, a high mortality rate and severe life-long sequelae in those who survive. The complement system is paramount for the innate immune defense against IMD, particulary by insertion of the bacteriolytic membrane attack complex into the bacterial membrane^3^. However, the pathogenic *N*. *meningitidis* express polysaccharide capsules which protect them against complement killing. The protection afforded by the *N*. *meningitidis* capsule can only be overcome by the host immune system by specific antibodies that target the complement system onto the bacterial surface via the classical pathway. These bactericidal antibodies are used as surrogate of protection by which efficacy of meningococcal vaccines is benchmarked^4^. A humoral response against *N*. *meningitidis* yielding bactericidal antibodies can be elicited either during asymptomatic colonization of the nasopharynx or by vaccination^5^. The lack of specific antibodies makes particularly infants and young children vulnerable to IMD, which is reflected by the age distribution of IMD incidence^6^.

IMD pathophysiology is the result of host responses to bacterial antigens activating multiple innate immune effector mechanisms upon uncontrolled *N*. *meningitidis* multiplication^7^. Most important aspects of IMD pathophysiology are the systemic inflammatory response syndrome (SIRS), disseminated intravascular coagulation (DIC) and vascular leakage leading to hypovolaemia, shock, multiorgan failure and, ultimately, death^8^. The events causing uncontrolled innate immune activation are under intense investigation in the hope to find therapeutic options adjunctive to immediate antibiotic treatment and fluid management, which specifically interfere with host inflammation in order to minimize mortality and sequelae. Here, the strict human-specific tropism of *N*. *meningitidis* is an obstacle for *in vivo* approaches to IMD, although several rodent infection models have been used successfully to recapitulate aspects of disease for the identifcation of new targets for intervention^9–13^.

As experimental *in vitro* approach, the whole blood infection model is propably the most valuable tool to investigate the interaction of *N*. *meningitidis* with its host during IMD, as it is relatively easy to implement, represents the correct host and features important consituents of cellular and soluble immune mediators relevant to IMD.

Indeed, whole blood models of IMD have been widely used in studies monitoring transcriptome dynamics of *N*. *meningitidis*^14–16^, the influence of meningococcal virulence factors^17–19^, the effect of complement factors^20,21^ and the impact of cellular effectors on IMD^22^.

However, the protocols for whole blood infections vary significantly among different studies. Of particular importance, different anticoagulants can be used to avoid blood clotting during the experiments. The most commonly used anticoagulants in studies regarding *N*. *meningitidis* whole blood infections are citrate^17,18^, heparin^14–16,23^ and hirudin (or its derivate lepirudin)^20,21,24,25^. These three anticoagulants inhibit coagulation by different mechansims: Citrate sequesters free Ca^2+^, a crucial co-factor of coagulation; the polyanionic glucosaminoglycan heparin inhibits coagulation mainly by enhancing the activity of antithrombin III; hirudin and its derivates directly bind to and irreversibly inhibit thrombin^26^. Chelating of Ca^2+^ not only inhibits coagulation, it also affects complement, a critical determinant in the defense against *N*. *meningitidis*. In fact, ethylenediaminetetraacetate (EDTA), a strong chelator of Ca^2+^ as well as Mg^2+^, blocks coagulation as well as complement entirely. A similar compound, ethyleneglycoltetraacetate (EGTA), displays a much lower affinity for Mg^2+^ than for Ca^2+^, and can be used to block coagulation as well as classical and lectin-pathway of complement activation.This divalent cation-dependent selectivity is a result of the critical Ca^2+^ dependence of the stability of the C1qr_2_s_2_ or MBL-MASP1/2 complexes, which initiate classical and lectin pathway, respectively. On the contrary, the alternative pathway can be initiated in presence of Mg^2+^ as the only divalent cation^27^. Thus, Mg/EGTA leaves the alternative complement pathway functionally intact^28,29^. Like EDTA and EGTA, citrate has an inhibitory effect on complement activation^30,31^. Heparin as well is an anticoagulant that intricately interferes with the complement system, with low doses (less than 2 units (U)/ml) reportedly activating and higher doses (from 20 U/ml) inhibiting complement^32^. In contrast to other anticoagulants, hirudin and its derivates are highly specific for thrombin and do not interfere with the complement system^21,32^.

The heterogeneity of anticoagulants used for whole blood models of *N*. *meningitidis* primed us to systematically analyze their actual impact on host cell responses as well as on meningococcal survival or growth and complement deposition, considering *N*. *meningitidis* isolates from carriers as well as from IMD cases.

## Results

### Influence of anticoagulants on innate immune responses during whole blood model of *N*. *meningitidis* infection

**Figure 1:**
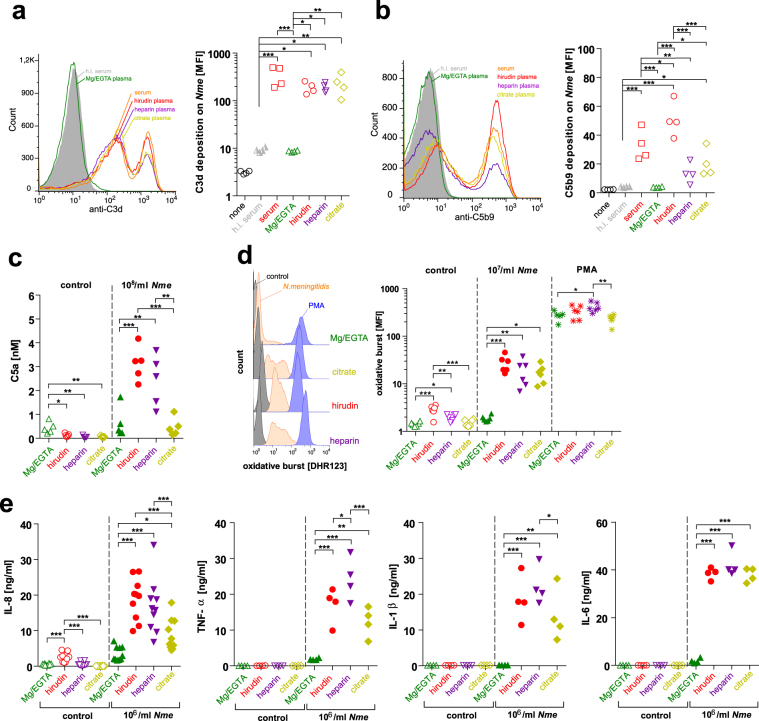
Impact of anticoagulants on innate immune responses during whole blood infection with *N*. *meningitidis*. (**a**) C3d fragment deposition and **(b)** C5b9 deposition on strain MC58 after 30 min incubation in 5% serum or plasma anticoagulated as indicated below x-axis. ‘None’ refers to control without serum or plasma but stained with the respective antibodies. Left graph shows representative flow cytometry histogram, right graph plots geometric mean fluorescence intensity (MFI). **(c)** C5a release as measure of overall complement activation after 30 min of *N*. *meningitidis* infection (10^8^ CFU/ml) with strain MC58 of whole blood anticoagulated as indicated below x-axis. Control refers to addition of RPMI medium instead of bacteria. **(d)** Oxidative burst response of neutrophils during infection with 10^7^ CFU/ml of strain MC58 after 1 h of infection of whole blood anticoagulated with the indicated anticoagulants. RPMI medium was added as negative control instead of bacteria (control), 1 µM phorbol-12-myristate-13-acetate (PMA) served as positive control. Left panel shows representative histograms, right panel plots mean fluorescence intensity of a total of 6 independent experiments (6 donors). **(e)** Release of inflammatory mediators IL-8, TNF-α, IL-1β and IL-6 in whole blood infected with 10^6^/ml of strain MC58 versus medium control after 4 h. All panels: *, **, *** indicates *P* < 0.05, 0.01 or 0.005 in one-way ANOVA applying Tukey’s *post hoc* test.

First, we analyzed the impact of different anticoagulants on the functional complement response towards *N*. *meningitidis* by incubating the bacteria with serum and plasma samples of immune donors (*i*.*e*. whose sera killed all meningococci upon 30 min of incubation). For anticoagulation, heparin, hirudin and citrate were compared, because they were used in previous studies of other researchers to demonstrate effects of *N*. *menigitidis* in whole blood models of infection. In addition, we also included Mg/EGTA, which inhibits coagulation and complement classical as well as lectin pathway, but leaves the complement alternative pathway intact. Deposition of C3d onto serogroup B *N*. *meningitidis* strain MC58 was similar in serum and plasma anticoagulated with hirudin, heparin or citrate, whereas it was entirely abrogated in Mg/EGTA (Fig. 1a). Interestingly, downstream assembly of the membrane attack complex (C5b9) on the bacteria varied significantly among the different anticoagulants: While hirudin plasma yielded slightly increased C5b9 deposition compared to serum, this was slightly reduced with citrate, strongly reduced with heparin and entirely abrogated with Mg/EGTA (Fig. 1b). As additional readout, whole blood infections were carried out and plasma C5a levels determined as indicator for overall complement activation. As shown in the left part of Fig. 1c, Mg/EGTA yielded weak but significantly elevated C5a-release compared to the other anticoagulants in absence of infection (‘control’). Indeed, an increased spontaneous activation of the alternative pathway has been noted before with Mg/EGTA^28^. When whole blood was incubated with *N*. *meningitidis*, significant C5a-levels were liberated, with highest concentrations in hirudin- and heparin-anticoagulated blood, and significantly less in citrate-anticoagulated blood. In Mg/EGTA anticoagulated blood, C5a levels were not elevated upon infection as compared to the uninfected control (Fig. 1c).

As readouts of cellular activation by *N*. *meningitidis* (strain MC58), the oxidative burst response of neutrophil granulocytes (PMN) was assessed using the DHR-123 assay (Fig. 1d) and the inflammatory chemokine IL-8 as well as the inflammatory cytokines TNF-α, IL-1β and IL-6 were measured in plasma after whole blood infection (Fig. 1e). Indeed, there was a notable anticoagulant-dependent influence on the cellular responses: The PMN oxidative burst response and the cytokine response towards *N*. *meningitidis* was almost entirely ablated in Mg/EGTA blood. In contrast, there was a robust oxidative burst response in infected hirudin-, heparin- or citrate-anticoagulated blood, with a (non-significant) trend to a lower signal with heparin and citrate as compared to hirudin (Fig. 1d). The cytokine/chemokine response was significantly lower with citrate (IL-8, TNF-α, IL-1β) than with hirudin or heparin, which in turn did not show a significantly different pattern, except for TNF-α, which was higher in heparin-anticoagulated blood (Fig. 1e). Of note, PMN were able to mount an oxidative burst in Mg/EGTA response upon stimulation with 1 µM PMA. Also, we noted that baseline activation of cellular responses during incubation at 37 °C without addition of bacteria was higher in hirudin and, to a lesser extent, also heparin anticoagulated blood as compared to Mg/EGTA or citrated blood.

### Anticoagulants influence *N*. *meningitidis* viability in BHI broth

We speculated that in addition to the host response, the anticoagulants might directly affect the bacteria. Thus, we considered differences in *N*. *meningitidis* viability in the presence of different anticoagulants by comparison of growth/survival curves of *N*. *meningitidis* in brain-heart-infusion broth (BHI) equipped with EDTA, EGTA, Mg/EGTA, heparin, hirudin or citrate in concentrations equivalent to those in whole blood after venipuncture using commercial blood collection tubes (*e*.*g*. Sarstedt Monovettes). In order to reflect the diversity within the species of *N*. *meningitidis* with respect to sequence types (ST) and invasiveness, we used seven strains of *N*. *meningitidis* from different clonal complexes (cc) isolated either from invasive cases (strain names are red in all display items) or asymptomatic carriers (strain names are blue in all display items), and we also included one strain of the closely related commensal *Neisseria lactamica*. An overview of the characteristics of the chosen strains is shown in Supplementary Table S1. We matched two pairs of carriage versus invasive strains which share high genomic homology in this study: MC58 (ST-74, cc32, IMD) versus α522 (ST-35, cc35, carriage)^14,33^ as well as 8013 (ST-177, cc18, IMD) versus α4 (ST-19, cc18, carriage)^34^.

The effects of the anticoagulants on *N*. *meningitidis* growth or survival in BHI broth are summarized in Table 1 and the corresponding growth curves are shown as Supplementary Figures S1 and S2. Evidently, there was a strong inhibitory effect on *N*. *meningitidis* growth when Ca^2+^ chelators EDTA or EGTA were present. In accordance with its high affinity for Mg^2+^ as well as Ca^2+^, growth inhibition was more pronounced with EDTA than with EGTA, which has considerably lower affinity for Mg^2+^ than for Ca^2+^. Equimolar addition of Mg^2+^ to EGTA (‘Mg/EGTA’) to restore overall concentration of free divalent cations significantly enhanced viability of most strains, but was not sufficient to allow for actual growth. The third calcium chelator, citrate, influenced meningococcal growth/survival to a markedly varying degree, depending on the strain. There was no obvious correlation between Mg/EGTA or citrate dependent growth inhibition and the carriage/disease phenotype of the *Neisseria* strains: Reduced growth in BHI with Mg/EGTA was observed for the matched strain pair MC58 (as well as its capsule mutant) and α522; however, only MC58 and its capsule mutant grew less well in presence of citrate. Of the other matched strain pair (α4 versus 8013), only α4 showed reduced CFU numbers after incubation with Mg/EGTA. Heparin and hirudin did not affect growth of the strains in BHI.

**Table 1:** Growth characteristics of *N*. *meningitidis* strains in BHI with added anticoagulants.

strain	SG	ST (cc)	BHI	EDTA	EGTA	Mg/EGTA	hirudin	heparin	citrate
α522	B	ST-35 (cc35)	**+++**	**+**	**+**	**+**	**+++**	**+++**	**+++**
MC58	B	ST-74 (cc32)	**+++**	**(−)**	**+**	**+**	**+++**	**+++**	**++**
DE9686	B	ST-42 (cc41/42)	**+++**	**+**	**+**	**++**	**+++**	**+++**	**++**
α4	B	ST-19 (cc18)	**+++**	**+**	**+**	**+**	**+++**	**+++**	**++**
8013	C	ST-177 (cc18)	**+++**	**−**	**+**	**++**	**+++**	**+++**	**+++**
α14	*cnl*	ST-53 (cc53)	**+++**	**−**	**+**	**++**	**+++**	**++**	**+**
MC58Δ*csb*	−	ST-74 (cc32)	**+++**	**−**	**+**	**+**	**+++**	**+++**	**++**
*N*. *lactamica* 020-06	−	ST-640 (cc640)	**+++**	**−**	**+**	**+**	**+++**	**+++**	**+**

SG, serogroup; ST, sequence type; cc, clonal complex; *cnl*, capsule null locus (no capsule). **+++** more CFU/ml at 6 h than at 0 h (overall growth). **++** steady levels of CFU/ml or growth after initial decline. **+** survival until 6 h. (**-**) survival in less than ½ of individual experiments. **-** no survival at 6 h.

### Anticoagulants influence *N*. *meningitidis* growth and survival in whole blood infection

**Figure 2:**
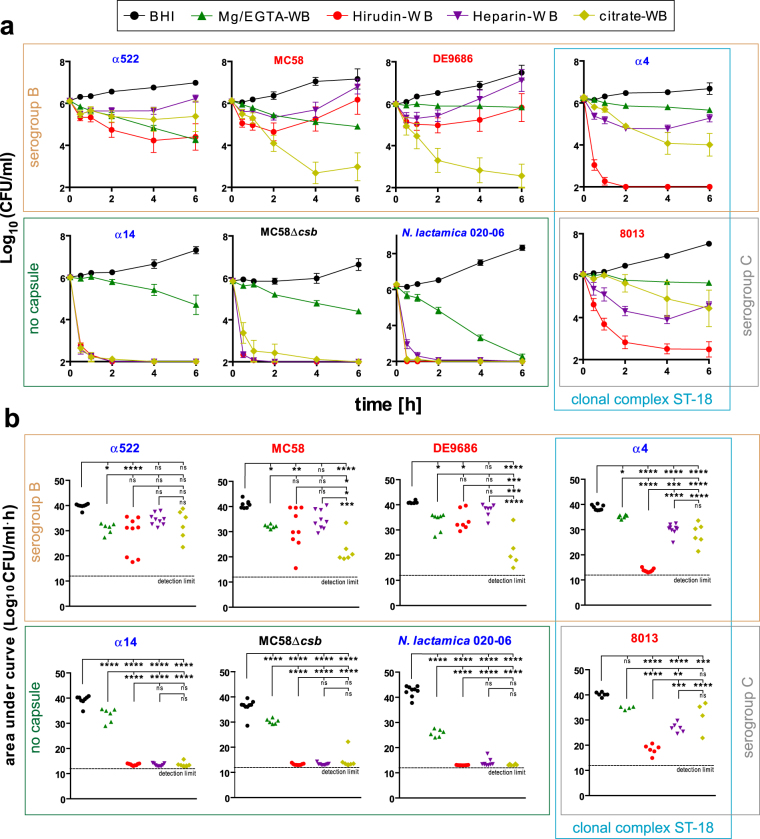
Influence of anticoagulants on survival of *N*. *meningitidis* strains (and *N*. *lactamica*) in whole blood infection. (**a)** Survival curves of various *N*. *meningitidis* strains and one *N*. *lactamica* strain (strain characteristics are in Supplementary Table S1; strains in red were isolated from IMD cases and strains in blue from asymptomatic carriers) in whole blood anticoagulated with different anticoagulants (see legend). Blood was drawn using monovettes containing the indicated anticoagulants from adult healthy donors with no history of meningococcal disease or vaccination and infected with 10^6^ CFU/ml of the indicated bacterial strains. At indicated time points, viable bacteria were enumerated after plating serial dilutions onto blood agar plates and plotted as log_10_ of CFU/ml. Limit of detection was 500 CFU/ml (log_10_ = 2.7), values below detection limit were plotted as 100 CFU/ml (log_10_ = 2). Plotted are means ± SEM of results from 6–9 independent experiments (donors). As positive control, bacteria were incubated in BHI instead of blood. **(b)** Area under curve analysis of the growth curves from the individual experiments (donors). The detection limit is calculated from the detection limit in (A) (log_10_ of CFU/ml set to 2 for negative samples) multiplied by 6 h. *, **, ***, **** denote *P* < 0.05, 0.01, 0.005, 0.0005 in one-way ANOVA applying Bonferrroni’s *post hoc* test. ns, not significant. Please note that the arrangement of plots, grouped by capsule characteristics and/or sequence type of the infection strain as highlighted by the coloured boxes, is continuous throughout this study.

In order to assess the influence of anticoagulants on *N*. *meningitidis* survival or growth in whole blood, we infected samples obtained from several non-immune donors using the four different anticoagulants Mg/EGTA, hirudin, heparin and citrate. The qualitative results of these experiments are summarized in Table 2, the corresponding growth curves are shown in Fig. 2a and the statistical analysis using the area under the curve is shown in Fig. 2b. For better access to the data, we grouped the strains by their capsule phenotype and/or genetic simiarity (by clonal complexes) as indicated by boxes in Fig. 2 and kept this organization for all figures of this study; further, we colour-coded the strains by their origin either blue (carriage isolates) or red (invasive disease isolates). The results indicate that the outcome of the whole blood infection depends on both, the *N*. *meningitidis* strain, as well as the anticoagulant. All strains showed logarithmic growth in BHI serving as positive control in each experimental run. All *N*. *meningitidis* strains, but not *N*. *lactamica*, survived until 6 h in whole blood anticoagulated with Mg/EGTA, showing a constant downside trend similar to that observed with BHI instead of blood (Supplementary Figure S2). Hence, while Mg/EGTA limits *N*. *meningitidis* growth in BHI, it also enables bacterial survival in whole blood by its inhibitory effect on the classical complement pathway^35^. This protection is particularly impressive for the *N*. *meningitidis* strains lacking capsule, which are rapidly killed in whole blood anticoagulated by any of the other anticoagulants. In heparinized whole blood, all encapsulated *N*. *meningitidis* strains survived and even showed growth after an initial drop in bacterial density. We presume that during this drop all meningococcal phase-variants that are not fit for survival in blood are eliminated whereas the remaining ‘fit’ variants propagate. On the contrary, when the direct thrombin inhibitor hirudin was used as anticoagulant, only strains α522, MC58 and DE9686 survived, whereas the two cc ST-18 strains bearing either serogroup B or C capsule did not. For all strains, the bacterial density over time was lower in hirudin-blood as compared to heparin-blood. Finally, survival of encapsulated *N*. *meningitidis* in citrate-blood was strongly strain-dependent, with low survival rates for MC58 and DE9686, which parallels to some extent the findings obtained with citrate in BHI. In addition, we did not see conclusive differences in strain survival as per disease/carriage origin of the strains; survival of *Neisseria* in blood was entirely dependent on the presence of capsule, though. Taken together, we found a strong influence of the anticoagulant on meningococcal survival in human whole blood, which is additionally dependent on the meningococcal strain.

**Table 2:** Survival and growth of *N*. *meningitidis* in whole blood using different anticoagulants.

strain	SG	ST (cc)	BHI	Mg/EGTA	hirudin	heparin	citrate
α522	B	ST-35 (cc35)	**+++**	**+**	**+**	**++**	**+**
MC58	B	ST-74 (cc32)	**+++**	**+**	**++**	**+++**	**(−)**
DE9686	B	ST-42 (cc41/42)	**+++**	**++**	**++**	**+++**	**(−)**
α4	B	ST-19 (cc18)	**+++**	**++**	**−**	**++**	**+**
8013	C	ST-177 (cc18)	**+++**	**++**	**(−)**	**++**	**+**
α14	*cnl*	ST-53 (cc53)	**+++**	**+**	**−**	**−**	**−**
MC58Δ*csb*	−	ST-74 (cc32)	**+++**	**+**	**−**	**−**	**−**
*N*. *lactamica* 020-06	−	ST-640 (cc640)	**+++**	**(−)**	**−**	**−**	**−**

SG, serogroup; ST, sequence type; cc, clonal complex; *cnl*, capsule null locus (no capsule). **+++** more CFU/ml at 6 h than at 0 h (overall growth). **++** steady levels of CFU/ml or growth after initial decline. **+** survival until 6 h. (**-**) survival in less than ½ of individual experiments. **-** no survival at 6 h.

### Hirudin-blood and hirudin-plasma closely reflect characteristics of *N*. *meningitidis* survival or killing in serum

**Figure 3:**
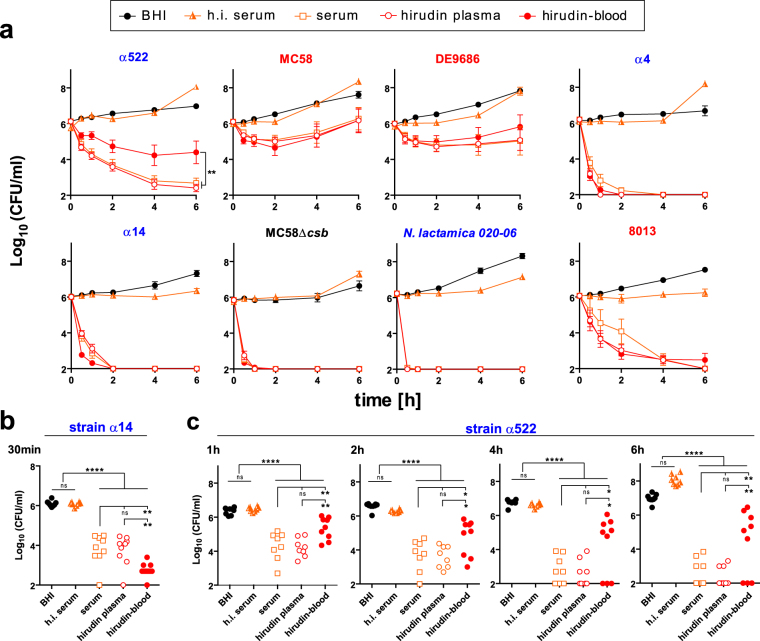
Comparison of *N*. *meningitidis* and *N*. *lactamica* survival in serum, hirudin plasma and hirudin blood. (**a)** Survival curves of the bacterial strains in serum, plasma or whole blood were conducted in parallel as those in Fig. 2. Heat-inactivated serum from the same donors was probed in a separate infection experiment to avoid delays in infection experiments of fresh whole blood samples. **(b)** Log_10_ of CFU/ml at the 30 min time point for strain α14 highlighting the significant differences between serum and hirudin-plasma versus hirudin-blood, in which clearance is faster. **(C)** Log_10_ of CFU/ml at the indicated time points (top left in each graph) for strain α522 highlighting the significant differences between serum and hirudin-plasma vesus hirudin-blood, in which the bacteria are partially protected from clearance. *, **, **** denote *P* < 0.05, 0.01, 0.0005 in one-way ANOVA applying Bonferrroni’s *post hoc* test. ns, not significant.

In order to analyze whether cellular mediators influence the rate of *N*. *meningitidis* viability or killing, we compared *N*. *meningitidis* growth/survival in hirudin-blood and hirudin-plasma with serum from the same donors. As shown in Fig. 3a, the survival curves of *N*. *meningitidis* in hirudin-blood and hirudin-plasma are almost identical to those in serum for five of the seven analyzed *N*. *meningitidis* strains, indicating no major impact of blood cells on their survival or killing. However, strain α14 is more rapidly cleared from hirudin-whole blood than in plasma or serum (Fig. 3b), which supports the notion that cellular components accelerate removal of this strain. On the contrary, strain α522 shows enhanced viability in whole blood as compared to hirudin-plasma or serum, indicating that blood cells have a protective effect on α522 (Fig. 3c). Thus, we observed three distict phenotypes: 1. Survival or killing independently of blood cells (5 of 7 strains); 2. Killing (partly) dependent on blood cells (α14); 3. Surivival dependent on blood cells (α522). Hence, we conclude that the influence of blood cells on *N*. *meningitidis* survival and their responses towards the bacteria can differ considerably for the individual strains.

### Heparin protects *N*. *meningitidis* against complement lysis in immune serum

**Figure 4:**
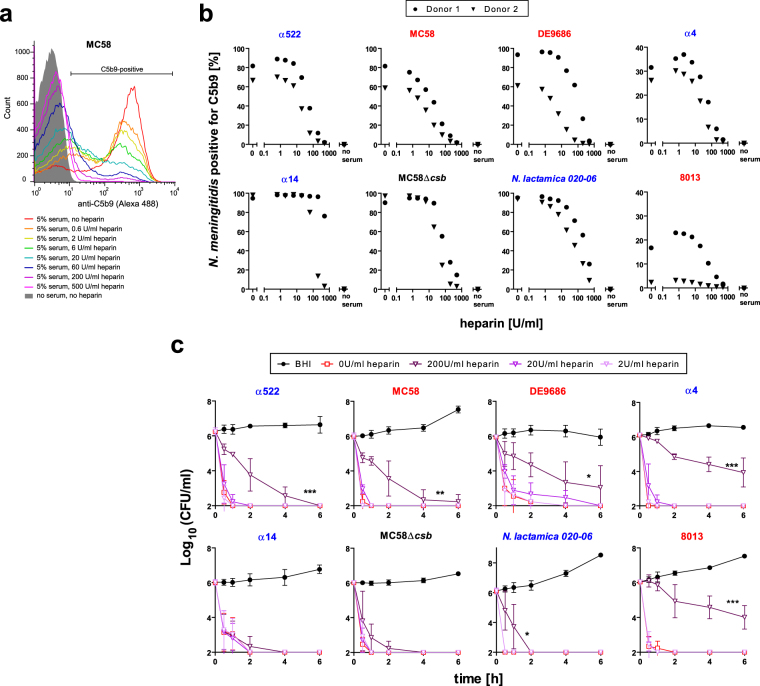
Influence of heparin concentration on C5b9 deposition and survival of *N*. *meningitidis* and *N*. *lactamica* in immune serum. (**a)** Representative histogram of C5b9 deposition onto strain MC58 incubated with 5% of immune serum in presence of different concentrations of heparin as indicated in legend. **(b)** Percentage of meningogocci positive for C5b9 as gated in histogram in (**a**) for the different *N*. *meningitidis* and *N*. *lactamica* strains plotted versus the heparin concentration as indicated under x-axis. Sera from two different donors in which no bacterial survival (all tested strains) in heparin-blood was found at 30 min were used as immune sera. **(c)** Survival curves of *N*. *meningitidis* and *N*. *lactamica* strains undiluted immune sera equipped with indicated concentrations of heparin versus BHI as positive control. Plotted are means ± SEM of three independent experiments (3 donors). *, **, *** denote *P* < 0.05, 0.01, 0.005 in one-way ANOVA applying Dunnett’s *post hoc* test versus ‘no heparin’ condition when comparing the area under the curve.

The consistently lower survival rates of encapsulated *N*. *meningitidis* in hirudin-blood than in heparin-blood (Fig. 2), together with its effects on C5b9-deposition onto the bacteria (Fig. 1b) primed us to examine the effect of heparin on bacterial survival in blood more closely. Heparin interacts with numerous cell surface proteins and plasma proteins including various factors of the complement system. In fact, heparin can inhibit complement activation to a considerable extent^36^, which is in agreement with the enhanced survival of *N*. *meninigitidis* in heparin-blood compared to that in hirudin-blood (Fig. 2). In order to assess the influence of heparin on complement deposition onto the bacteria, we incubated the *N*. *meningitidis* strains with sera from two immune individuals in presence of different concentrations of heparin and measured deposition of membrane attack complex (C5b9) by flow cytometry (Fig. 4a,b). Indeed, there is a dose-dependent reduction of C5b9-deposition by increasing concentrations of heparin. In addition, we tested whether addition of heparin to immune sera facilitates *N*. *meningitidis* survival (Fig. 4c). As a matter of fact, addition of high concentrations of heparin (20–200 U/ml) enabled survival of the encapsulated meningococcal strains even in undiluted immune sera. As a reference to put the heparin concentrations used here into biological context, the final concentration of heparin in whole blood drawn with heparin-monovettes is 19 U/ml; normal (endogenous) plasma heparin levels have been reported at 0.1–0.24 U/ml (1.0–2.4 µg/ml)^37^, or 1.9 ± 1.7 U/ml^38^, or 0.1–0.2 U/ml^39^, or 0.5 U/ml (5.4 ± 1.7 µg/ml)^40^. These values have to be used with caution, though, since the definition of one unit (U) has changed over time and can vary between different countries (*e*.*g*. USP units and IU vary by 7–13%); additional uncertainty stems from the different methods of measuring heparin anticoagulant activity. Patients undergoing cardiopulmonary bypass receive a bolus dose of ~300 U/kg heparin, resulting in a theoretical concentration of 3–5 U/ml blood (calculated with an approximate 70 ml blood per kg body weight). It must be noted, though, that heparin as a component of the extracellular matrix is sequestered onto the surface of cells and it is in equilibrium between the cell-bound and the free state. For endothelial cells and extracellular matrix, the dissociation constant of heparin binding is about 1 µM^41^.

### Heparin binding to the *N*. *meningitidis* surface is not required for protection in immune serum

**Figure 5:**
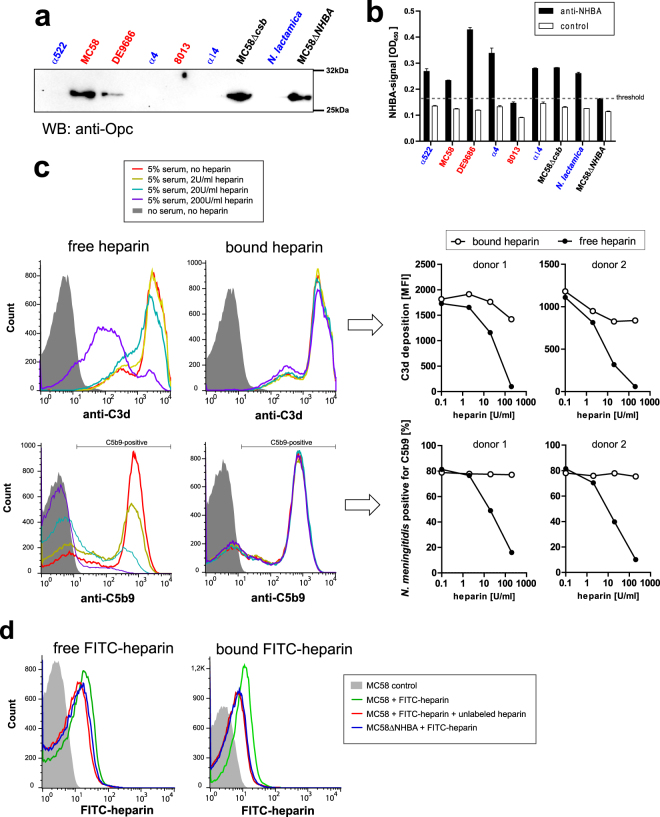
Interaction of heparin with *N*. *meningitidis* surface proteins is not required for protection against complement deposition. (**a**) Western blot for detection of Opc expression by bacterial strains. The uncropped image is depicted in Supplementary Figure S4. (**b**) Whole-cell ELISA for detection of NHBA expression by bacterial strains. (**c**) Left panels: Representative histograms of C3d (upper graphs) or C5b9 (lower graphs) deposition onto strain MC58 incubated in 5% immune serum. In the ‘free heparin’ condition, heparin was added to the bacteria concommittantly with the immune serum; in the ‘bound heparin’ condition, bacteria were first incubated with heparin then washed and then the immune serum added. Heparin concentrations are indicated in the legend. Right panels: Mean fluorescence intensity of C3d deposition (upper graphs) or percentage of *N*. *meningitidis* with C5b9 deposition (lower graphs) as in histograms in left panels. The experiments were carried out with immune sera from 2 immune donors (same as in Fig. 4). (**d**) Binding of heparin to *N*. *meningitidis* as determined by flow cytometry using FITC-labeled heparin at 100 µg/ml following the same protocol for ‘free’ and ‘bound’ heparin as in (**c**). Specificity of FITC-labeled heparin binding is demonstrated by partial displacement by a 20-fold excess of unlabeled heparin; Further, binding of FITC-heparin to MC58ΔNHBA was monitored.

Since *N*.*meningitidis* utilizes heparin for cellular adhesion and invasion via its opacity protein (Opc)^42^ and its Neisseria heparin binding antigen (NHBA)^43^, we hypothesized that these factors might recruit heparin onto the surface of *N*. *meningitidis* to locally regulate complement activation. First, we tested whether the meningococcal strains expressed either of the two heparin binding proteins. Western blot analysis identified only strains DE9686 as well as MC58 and its derivates to be Opc-positive (Fig. 5a), whereas NHBA expression was found in all strains except 8013 and MC58ΔNHBA by whole-cell ELISA (Fig. 5b). In order to test whether cell-bound or free heparin is responsible for protection against complement, we compared two conditions for strain MC58: Either the bacteria were incubated with different concentrations of heparin, then unbound heparin was washed away before addition of 5% immune serum (‘bound heparin’), or the bacteria were incubated with heparin and serum without washing (‘free heparin’). After incubation at 37 °C for 30 min, the reaction was stopped and deposition of C3b (by C3d fragment) and C5b9 was analyzed by flow cytometry. As shown in Fig. 5c, protection only occurs in the ‘free heparin’ condition. In order to verify that heparin stays surface bound after the wash steps in the ‘bound’ condition, *N*. *meningitidis* were incubated under the same conditions with fluorescein-heparin (100 µg/ml) and subsequently either washed or not washed as above before analysis by flow cytometry. Indeed, fluorescein-heparin bound to the bacteria and was detectable after the wash steps. Furthermore, fluorescein-heparin binding could be at least partially competed out by addition of a ~20-fold excess (2000 µg/ml) of unlabeled heparin (Fig. 5d). Accordingly, MC58ΔNHBA bound less FITC-heparin as the wildtype strain. Therefore, we conclude that a direct interaction between heparin and *N*. *meningitidis* is not required for protection in our assay. This claim is also supported by the fact that strain 8013, devoid of NHBA and Opc, shows enhanced survival in heparin blood (Figs 2, 4).

Since NHBA is a component of the 4-component meningococcal serogroup B vaccine (Bexsero), we considered the impact of this surface-antigen on C5b9-deposition and survival in immune sera in presence or absence of heparin. When compared to the parental strain (MC58), *N*. *meningitidis* lacking NHBA (MC58ΔNHBA) displayed a non-significant trend towards reduced levels of C5b9 deposition after incubation with immune sera in absence of heparin. However, both strains showed significant reduction of C5b9 deposition in presence of 20 U/ml heparin (Supplementary Figure S3a). Furthermore, in presence of 200 U/ml heparin, MC58ΔNHBA showed significantly higher survival in undiluted immune sera as compared to the parental strain (Supplementary Figure S3b).

Thus, NHBA seemingly does not have a net negative effect on complement deposition onto the bacteria in immune sera and it does not appear to be significant for the heparin-induced complement inhibition in immune serum. We conclude that NHBA is not involved in serum resistance through its heparin binding capability. Although we lacked the ability to measure NHBA-specific antibodies in the immune sera used, we speculate that the immune sera used here contained antibodies against NHBA for two reasons: Either individuals had previouyls received the 4-component meningococcal serogroup B vaccine, which induces robust anti-NHBA antibodies^43^, or the subjects have acquired natural immunity by nasopharyngeal carriage, which as well can generate anti-NHBA antibodies^44^. Thus, it is possible that antibody binding to NHBA yielded a higher deposition of complement onto the wildtype bacteria than onto the mutants. This effect could have potentially masked any complement evasion effect through the NHBA protein.

### Excess heparin limits factor H binding to *N*. *meningitidis*

**Figure 6:**
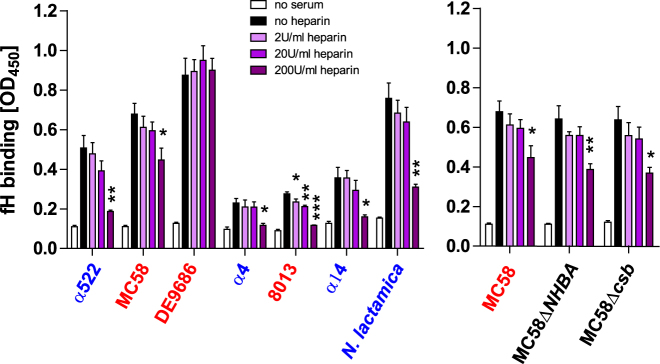
Influence of heparin on factor H binding to *N*. *meningitidis* in human serum. ELISA plates were coated with heat-killed bacteria and incubated with 5% heat-inactivated serum samples equipped with indicated concentrations of heparin. *N*. *meningitidis* bound fH was detected using anti-fH antibody. Plotted is the mean ± SEM of three independent experiments, serum from a different donor used in every experiment. *, **, *** denote *P* < 0.05, 0.01, 0.005, respectively, in one-way ANOVA applying Dunnett’s *post hoc* test comparing the different heparin concentrations with the ‘no heparin’ condition.

*N*. *meningitidis* recruits the complement regulator protein factor H (fH) via fHbp, NspA or sialylated LOS to their surface to resist complement deposition^45–47^. On the other hand, heparin can bind to fH as well^48^. Since heparin can interact with *N*. *meningitidis* through Opc or NHBA surface protein, we wondered whether the presence of heparin enhances fH binding to *N*. *meningitidis*. Using a whole-cell ELISA, we found that heparin does not enhance serum fH binding to the bacteria, but rather the opposite: At higher heparin concentrations, fH binding to all analyzed strains is significantly reduced, except for strain DE9686, which displayed the strongest binding to fH among our collection of strains (Fig. 6). Notably, MC58 parental strain and its derivative lacking NHBA showed the same result, further supporting that interactions of heparin with this surface protein does not impact on fH binding.

## Discussion

Our study demonstrates that both, the *N*. *meningitidis* strain as well as the choice of anticoagulant can influence the results of whole blood infections with *N*. *meningitidis*. Firstly, there is a direct influence of the anticoagulant on the bacteria, which affects individual strains to a different extent. Secondly, the anticoagulants Mg/EGTA, heparin and citrate dampen the complement activation to a different overall extent and at distinct stages of the complement cascade. Thirdly, different anticoagulants affect cellular responses of the innate immune system to a somewhat varying extent, and also depending on the functional readout (*e*.*g*. oxidative burst, cytokine release). Thus, a thorough testing of the appropriateness of anticoagulant and the choice of *N*. *meningitidis* strain in the context of the experimental goal is advised when using the whole blood infection model.

The appropriateness of anticoagulants in human whole blood models of IMD has been analyzed before to some extent by other researchers. A study conducted by Ison *et al*. found that survival of serogroup A, B and C as well as non-capsule *N*. *meningitidis* in whole blood depended on both, the anticoagulant and the strain, which is in good agreement with our study^49^. Further, they found that release of TNF-α was not strongly influenced by anticoagulant or strain, but that PMN degranulation was affected by the anticoagulant. However, experiments conducted in the study by Ison *et al*. were only for a 1 hour duration, compared only citrate (3.8% w/v) and heparin (10 U/ml) and the blood was only from one single donor, which had been immunized several years before the study with a serogroup ACWY polysaccharide vaccine.

Mollnes *et al*. were the first to suggest the hirudin derivative lepirudin (Refludan^®^) as anticoagulant for whole blood infection models of *E*. *coli*^32^. They compared complement activation in whole blood anticoagulated with different concentrations of lepirudin versus heparin, showing that while heparin partially inhibits complement, hirudin does not^32^. Thus, lepirudin is regarded the best choice of anticoagulant for any whole blood assay for infections in which complement mediated effects are crucial, which definitely includes *N*. *meningitidis*^21^. Lepirudin differs from hirudin by a leucin residue at position 1, a threonin residue at position 2 and lack sulfation at tyrosin at position 63.

A further study conducted by van der Maten and co-workers elucidated the effects of numerous anticoagulants on whole blood models of *S*. *pneumoniae*^50^. Here, the superiority of hirudin over other anticoagulants was noted because it did not interfere with pathogen opsonization by C3b which is particularly important for *S*. *pneumoniae* clearance from blood. Futher, the authors conducted experiments confirming that complement-mediated killing of *N*. *menigitidis* in whole blood is observed with hirudin as anticoagulants and dependent on functional C6^50^.

In general, our study is in very good agreement with the above mentioned studies, indicating that the anticoagulant can have a significant impact on whole blood infection models and that hirudin or its derivatives are superior over heparin or citrate (or Mg/EGTA). However, no study has yet considered either the breadth of meningococcal isolates covering isolates from carriers or disease, encapsulated versus non-capsulated phenotypes or different sequence types. In addition, the direct influence of anticoagulants on *N*. *meningitidis* growth and survival has not been described before, which we found to significantly differ among the different substances and also among different *N*. *meningitidis* strains. Also, no study has yet directly compared side-by-side the influence of all three relevant anticoagulants (citrate, heparin, hirudin) during *N*. *meningitidis* infection of whole blood.

We did not see a conclusive difference in whole blood survival or growth of meningococcal strains as per the circumstances of their isolation from either asymptomatic carriers or patients suffering from IMD (Fig. 2). Not surprisingly, the presence of capsule defined the ability of *N*. *meningitidis* to survive in whole blood. The cc18-strain pair of α4/8013 showed a very similar outcome in our whole blood infection assays, both being susceptible to killing in hirudin-blood but survival and growth in heparin-blood, despite their different phenotypes (carriage versus IMD isolate) and serogroup (B versus C). The other strain pair, MC58/α522 showed no differences in growth or survival in hirudin- or heparin-whole blood, but differed in their survival in citrate-whole blood and serum or hirudin-plasma (Fig. 3). Thus, although our study only takes into account a small number of strains, it appears that survival and growth of *N*. *meningitidis* strains is not solely determined by their serogroup or overall genetic similarity as per sequence type, which is in line with the notion that meningococcal virulence is a polygenic trait and that the characterization of distinct genetic elements responsible for their invasiveness are obscured by the high genetic diversity of this species^14,33,51^.

In addition to citrate, heparin and hirudin, we also considered Mg/EGTA as possible anticoagulant, which allows the differentiation of the impact of the alternative complement pathway versus the classical and lectin pathway^28,29^. We found that all *N*. *meningitidis* strains (but not *N*. *lactamica*) survived in Mg/EGTA blood, even when lacking capsule (Fig. 2). While it is clear that the alternative pathway alone is not sufficient to kill encapsulated *N*. *meningitidis*, enabling them to survive in blood or serum from naive donors, we were surprised that the alternative pathway alone was insufficient to kill non-capsule *N*. *meningitidis* strains. In fact, the survival curves of all bacterial strains were nearly identical in growth medium (BHI) or whole blood equipped with Mg/EGTA (Fig. 2, Supplementary Figure S2). This underscores that the alternative pathway, although crucial for the complement mediated killing of *N*. *meningitidis*^3,52^, primarily aids in bacterial removal by amplifying antibody-dependent classical pathway activation, which is in agreement with the notion that the meningococcal capsule primarily inhibits classical pathway activation^53^. It was not surprising though, that the use of Mg/EGTA by virtue of its Ca^2+^ chelating capacities also inhibited leukocyte activation by *N*. *meningitidis* (Fig. 1), presumably by interfering with Ca^2+^ dependent intracellular signalling transduction. Thus, in addition to complement activation, *N*. *meningitidis* killing by cellular effectors in whole blood is also likely hampered by Mg/EGTA in whole blood.

EDTA, EGTA and citrate are all chelators of divalent cations such as Ca^2+^ and Mg^2+^. While EDTA inhibits complement entirely, EGTA, due to its lower affinity for Mg^2+^ inhibits only the classical and lectin pathway. Citrate as well is a Ca^2+^ chelator, and partially inhibits complement activation by *N*. *meningitidis* (Fig. 1), but it has much less inhibitory activity on complement than EGTA. The different potencies of EDTA, EGTA and citrate to inactivate complement are explained by their different affinities for either Ca^2+^ or Mg^2+^: Citrate only weakly binds Ca^2+^ and very weakly Mg^2+^ (K_D_ of 16 µM and 1,6 mM, respectively), while EGTA binds Ca^2+^ extremely strongly, but Mg^2+^ only weakly (K_D_ of 13 pM and 4 µM, respectively); EDTA binds Ca^2+^ as well as Mg^2+^ extremely strongly (K_D_ of 20 pM and 2 nM, respectively). The same argument holds true for all cellular activation patterns observed, which differ between Mg/EGTA and citrate, such as oxidative burst and cytokine/chemokine response (Fig. 1).

Different studies agree that hirudin (or its derivates) is superior over other anticoagulants in infection assays where complement is important;^21,32,50^ however, there are considerable limitations in the use of hirudin or its derivatives. As evident from data in Fig. 1, hirudin anticoagulated blood results in a significantly elevated background signal in neutrophil responses (oxidative burst) and IL-8 secretion, despite the fact that blood was taken by experienced personnel and handled carefully and used immediately (within 10 min after the draw) for our assays. We assume that low grade complement activation is not counterbalanced when no complement inhibitory activity is present (as observed with all other anticoagulants) during the time of incubation at 37 °C in the assays. Hence, longer incubation periods will inevitably yield cellular activation to some extent. However, we do not consider this to be a major obstacle, since whole blood infection models *per se* only permit assays to be conducted within a relatively short time frame of less than a few hours. A further limitation is that production of lepirudin (Refludan^®^), which is most extensively used by most researchers^21,22,25,32^, has been discontinued in 2012 and markets are entirely depleted from pharmacologic-grade commercially available lepirudin. This means that alternatives are required, since the stockpiles which are currently in use have already reached their shelf-life and will be used up in the near future. A suitable alternative to lepirudin is desirudin, another variant of hirudin that lacks a sulfate group at tyrosine 63, which is available in North America (Iprivask^®^) but it is no longer licenced in Europe (Revasc^®^). However, the use of desirudin has not been broadly established for whole blood infection models yet. We found that the other direct thrombin inhibitors argatroban and bivalirudin (the latter one being a synthetic peptide derived from hirudin) were not suitable as replacements of lepirudin or hirudin in the whole blood infection model, as they do not bind irreversibly to thrombin^54^ and do not completely inhibit blood clotting *ex vivo* within the time frame of our experiments even at high concentrations. In this study, we used hirudin monovettes (obtained from Sarstedt) which we found to be suitable for whole blood infections, but feature only low blood volumes to be drawn per monovette (currently 1.6 ml).

Our data indicate that heparin enhances *N*. *meningitidis* survival in blood (Fig. 2), which was particularly striking in blood of immune donors at high concentrations of heparin (Supplementary Figure S4). Although low concentrations of heparin (≤5 U/ml) have been reported to activate complement *in vitro*^32^, we found that C5b9 deposition onto *N*. *meningitidis* is slightly reduced in presence of as little as 2 U/ml (Fig. 4b). In this context it is tempting to speculate about the effect of heparin treatment of patients with meningococcal sepsis in terms of protection of *N*. *meningitidis* against complement^55–57^. However, any inhibitory effect of heparin on the complement system would not be relevant to survival of the bacteria, which are rapidly killed upon the immediate administration of antibiotics to the patients. It is worth mentioning, though, that inhibition of certain aspects of complement such as the pro-inflammatory C5a and its signaling receptor, C5aR, can indeed improve outcomes of experimental IMD^13^.

Our data indicate that the mode of action of heparin in protecting *N*. *meningitidis* against complement-mediated lysis is independent of the capability of the bacteria to bind heparin by Opc or NHBA. In our strain collection, only DE9686 as well as MC58 and mutants thereof were positive for Opc, whereas all strains except 8013 and MC58ΔNHBA expressed NHBA (Fig. 5a,b). Strain MC58, which expresses both, Opc and NHBA, displayed no reduction of C5b9 deposition, when the bacteria were first incubated with heparin and then washed before addition of immune sera (Fig. 5c). In addition, heparin appears to compete with the *N*. *meningitidis* fH-sequestration moieties for fH, effectively reducing *N*. *meningitidis*-bound fH at high concentrations, and this was independent of NHBA (Fig. 6). Hence there does not seem to be a functional role of Opc or NHBA in protection against complement via heparin binding, in addition to their established roles as adhesins and invasins^43,58^. Interestingly, *N*. *meningitidis* strain MC58 lacking NHBA showed increased survival in immune sera as compared to the wildtype strain when 200 U/ml heparin were present (Figure S3b). We were not able to determine implications of anti-NHBA antibodies in the immune sera in this assay, but together with the fact that NHBA is upregulated at low temperatures as found in the nasopharynx^59^ and dowregulated at temperatures above 37 °C (*i*.*e*. fever)^60^, it supports the notion that NHBA is primarily a factor required for mucosal colonization but seems less advantageous for *N*. *meningitidis* during blood stream infection.

In summary, caution is advised when conducting whole blood infections, as the chosen anticoagulant as well as the individual strain of *N*. *meningitidis* can significantly impact on the results.

## Methods

### Bacteria

*N*. *meningitidis* and *N*. *lactamica* strains were grown on Columbia sheep agar plates (BioMerieux) at 37 °C, 5% CO_2_ and water saturated atmosphere. Detailed information on the bacterial strains used in this study can be found in the Supplementary Table S1. To prepare inocula for experiments, bacteria were resuspended in RPMI medium (Life Technologies) and adjusted to an OD_600_ of 1.0, which corresponded to 10^9^ CFU/ml for all strains, except for *N*. *lactamica*, which was adjusted to OD_600_ of 2.0 to yield the same density. Serial dilutions were made in RPMI medium to obtain the required bacterial density for infection experiments; bacterial inocula were always verified by plating and colony enumeration.

### Whole blood model

Whole blood was collected by venipuncture of adult healthy volunteers not taking antibiotics or anti-inflammatory medication within the last 14 days and without any known history of meningococcal disease or vaccination. The study protocol was approved by the Ethics Committee of the Medical Faculty of the University of Würzburg (file 181/16-ge) and was carried in accordance with the relevant local guidelines and in compliance with the Helsinki Declaration. All participants (donors) expressed their informed consent in written form. Blood was drawn using the following monovettes (all from Sarstedt): K_3_EDTA (02.1066.001, final concentration 1,6 mg/ml = 5.5 mM), Li-heparin (01.1604.001, final concentration 19 U/ml), Na_3_citrate (05.1071, final concentration 106 mM), hirudin (04.1959.001, final concentration 525 antithrombin units/ml), Serum-monovette (02.1063). For EGTA, syringes were aseptically filled with sterile EGTA adjusted to pH7.4, either with or without addition of an equimolar amount of MgCl_2_ to yield 10 mM final concentration. Infection assays for monitoring bacterial survival or growth were carried out in 100 µl volume in sterile, pyrogen-free 0.2 ml reaction tubes at a final bacterial density of 10^6^ CFU/ml, a density which is observed in human patients suffering from meningococcemia. Samples were incubated at 37 °C rotating over top at 20 rounds per min to avoid sedimentation of blood cells. At indicated time points, 5 µl samples were taken and serially diluted in Dulbecco’s phosphate buffered saline (PBS; Sigma-Aldrich) and plated onto Columbia sheep blood agar plates (BioMerieux) for CFU enumeration. As a positive control in every experimental run, brain heart infusion (BHI) was used to monitor bacterial growth; another positive control was heat-inactivated (56 °C for 30 min) serum of the same donor.

### Determination of inflammatory cytokines and chemokines

Infection of whole blood samples was carried out as described above at a final density of 10^6^ CFU/ml of bacteria and incubated for 4 h at 37 °C rotating over top. After 4 h cellular responses were halted by addition of 100 µl of ice-cold PBS with 40 mM EDTA, samples were centrifuged at 4000 g for 5 min at 4 °C, supernatant was collected and shock-frozen on dry ice until IL-8 ELISA (DouSet^®^ ELISA kit, R&D Systems)was performed according to the manufacturer’s instructions. Additional cytokines (TNF-α, IL-1β and IL-6) were measured in a separate experiment using the bead-based LegendPlex assay according to the manufacturer’s instructions (human inflammation kit, Biolegend).

### Oxidative burst assay

For measurement of the oxidative burst, 75 µl blood samples in 1.5 ml reaction tubes were mixed with dihydrorhodamine (DHR)-123 (Sigma-Aldrich), a oxidation-sensitive dye, at a final concentration of 6 µM. Then, bacteria were added at a final density of 10^7^/ml. Instead of bacteria, RPMI was added in negative controls or PMA at 1 µM (final concentration) in RPMI was added in positive controls. Samples were incubated at 37 °C rotating over top for 1 h and then 1050 µl of ice-cold erythrocyte lysis buffer (168 mM NH_4_Cl, 10 mM KHCO_3_, 125 µM EDTA, pH 7.3) was added and tubes inverted for approximately 5 min until erythrocytes were visibly lysed. Then, samples were centrifuged at 10,000 g for 5 min at 4 °C, supernatant with erythrocyte ghosts carefully removed and cell pellet washed with 1 ml of PBS. After centrifugation as before, the washed pellet was fixed with 4% formaldehyde in PBS for 30 min before analysis. Flow cytometry was done with a FACScalibur (Becton Dickinson), data analysis was performed using FlowJo v10 software (FlowJo). Polymorphonuclear cells were gated by their prominent FSC/SSC characteristics and DHR-123 fluorescence was recorded.

### Flow cytometry for complement deposition on bacteria

Bacteria were harvested from Columbia sheep blood agar plates and adjusted to OD 0.1 in complement binding reaction buffer (KBR-buffer: 10 mM HEPES, 125 mM NaCl, 1 mM MgCl_2_, 1 mM CaCl_2_, pH 7.3; Virion\Serion) with 0.1% bovine serum albumin (BSA; Applichem). In a conical 96well plate (Sarstedt), 10 µl of heparin (Biochrom) dilutions were mixed with 100 µl of bacteria and finally 5.6 µl (5% final volume) serum or plasma from immune human subjects was added. Where indicated (Fig. 5), bacteria were first incubated with heparin for 30 min, then unbound heparin washed away with KBR, 0.1% BSA, before addition of serum. Complement deposition was allowed for 30 min at 37 °C, then stopped by addition of 150 µl of ice-cold PBS, 1% BSA, 10 mM EGTA, 10 mM MgCl_2_. The plate was centrifuged 5 min at 4000 g, 4 °C, supernatant removed and pellets washed with 180 µl of ice-cold PBS and the plate centrifuged again. Then, pellets were resuspended in 50 µl of PBS, 0.1% BSA containing either 1 µl mouse-anti-C5b9 clone aE11 (Dako M0777) or polyclonal rabbit-anti-C3d (Dako 0063). Samples were incubated on ice for 1 h, then washed as above and pellets resuspended in 50 µl of PBS, 0.1% BSA before 1 µl of secondary antibody was added (goat-anti-mouse-Alexa 488 (Jackson ImmunoResearch 115-545-166) or goat-anti-rabbit-Alexa 488 (Jackson ImmunoResearch 111-545-144)). After 1 h on ice, samples were washed again with PBS, bacteria fixed with 4% formaldehyde solution in PBS for 30 min before analysis on a FACScalibur. Data were analyzed with FlowJo v10.

### Flow cytometry for FITC-heparin binding to *N*. *meningitidis*

*N*. *meningitidis* strain MC58 or its isogenic mutant lacking NHBA (MC58ΔNHBA) was harvested from overnight growth on Columbia sheep blood agar plates and resuspended in KBR buffer (Virion\Serion) with 0.1% BSA and adjusted to OD 0.1. In a conical 96well plate (Sarstedt), 10 µl of FITC-heparin (ThermoFisher H7482) was added to 100 µl bacterial suspension, resulting in 100 µg/ml FITC-heparin. Where indicated, a 20-fold excess of heparin (2000 µ/ml final concentration; Biochrom) was added. Samples were incubated 30 min at room temperature. For ‘bound FITC-heparin’, samples were washed as above with cold KBR + 0.1% BSA, whereas washing was omitted for ‘free FITC-heparin’ samples. Bacteria were pelletted and resuspended in 4% formaldehyde solution in PBS for 30 min before analysis on a FACScalibur flow cytometer.

### Western blot

Bacteria were resuspended in phosphate buffered saline (PBS) at OD 6,0, lysed by addition of one volume of sample buffer (125 mM Tris pH 6.8, 4% (w/v) SDS, 20% glycerol and 10% β-mercaptoethanol) and samples boiled for 10 min prior to analysis on a 10% SDS polyacrylamid gel and blotting onto nitrocellulose. Blots were stained using anti-Opc antibody clone B306^61^ followed by goat-anti-mouse-HRP conjugate (Jackson ImmunoResearch 115-035-003). Bands were visualized by addition of enhanced chemiluminescence reagent (Bio-Rad) and imaged using a Chemi Doc Imaging System (Bio-Rad).

### Whole cell ELISA for NHBA

*N*. *meningitidis* suspension in PBS was adjusted to OD 0.1, heat-inactivated at 65 °C for 30 min and 50 µl per well of 96-well microtitre plates (Sarstedt) let to dry overnight. The plates were washed thrice with wash buffer (PBS + 0.05% Tween-20), blocked with 5% bovine serum albumin (BSA) before addition of polyclonal rabbit anti-NHBA antibody. Detection was done with goat-anti-rabbit IgG-horseradish peroxidase conjugate (Jackson ImmunoReserach 111-035-144) and TMB substrate (Thermo Fisher Scientific).

### Whole cell ELISA for fH

*N*. *meningitidis* coated microtitre plates were washed and blocked as above. Then, 5% human serum from 4 different non-immune donors along with the indicated concentrations of heparin (Biochrom) were added and incubated for 1 h at room temperature before washing thrice. *Nme-*bound fH was detected by addition of goat-anti-fH antibody (CompTech A237) followed by donkey-anti-goat-HRP conjugate (Jackson ImmunoResearch 705-035-003) and TMB (Thermo Fisher Scientific) readout.

### Statistic analysis

Data were plotted and statistically analyzed using GraphPad Prism. The test used and significance statements (as per *P*-value) are indicated in the respective figure legends. Generally, comparisons of more than two groups were conducted using one-way ANOVA with appropriate *post hoc* tests as indicated.

### Data availability

All data and materials associated with this publication are available upon request.

## Electronic supplementary material

supplementary information
