# Supplementary material for: Anticoagulants impact on innate immune responses and bacterial survival in whole blood models of Neisseria meningitidis infection
Source: Sci Rep. 2018 Jul 5;8:10225. doi: 10.1038/s41598-018-28583-8 (PMC6033889; doi:10.1038/s41598-018-28583-8)
Supplement: Supplementary file 1 — supplementary information [file 41598_2018_28583_MOESM1_ESM.pdf]

Supplementary information to

Anticoagulants impact on innate immune responses and bacterial survival in whole blood models of *Neisseria meningitidis* infection

Lea Strobel [1] and Kay O. Johswich[1]*

[1]: Institute for Hygiene and Microbiology, University of Wuerzburg, Wuerzburg, Germany

Supplementary table S1: Characteristics of bacterial strains used in this study

strain	species	serogroup (capsule)	sequence type (clonal complex)	invasive/ carrier	reference
α522	***N. meningitidis***	**B**	**ST-35 (cc35)**	carrier	1
MC58	***N. meningitidis***	**B**	**ST-74 (cc32)**	invasive	2
DE9686	***N. meningitidis***	**B**	**ST-42 (cc41/44)**	invasive	3
α4	***N. meningitidis***	**B**	**ST-19 (cc18)**	carrier	1
8013	***N. meningitidis***	**C**	**ST-177 (cc18)**	invasive	4
α14	***N. meningitidis***	**-**	**ST-53 (cc53)**	carrier	1
**MC58Δ*csb***	***N. meningitidis***	**-**	**ST-74 (cc32)**	**(mutant)**	5
**MC58ΔNHBA**	***N. meningitidis***	**B**	**ST-74 (cc32)**	**(mutant)**	6
020-06	***N. lactamica***	**-**	**ST-640 (cc640)**	carrier	7

1 Claus, H., Maiden, M. C., Maag, R., Frosch, M. & Vogel, U. Many carried meningococci lack the genes required for capsule synthesis and transport. *Microbiology* **148**, 1813-1819 (2002).

2 McGuinness, B. T. *et al.* Point mutation in meningococcal por A gene associated with increased endemic disease. *Lancet* **337**, 514-517 (1991).

3 Elias, J. *et al.* Vaccine preventability of meningococcal clone, Greater Aachen Region, Germany. *Emerging infectious diseases* **16**, 465-472, doi:10.3201/eid1603.091102 (2010).

4 Nassif, X. *et al.* Antigenic variation of pilin regulates adhesion of *Neisseria meningitidis* to human epithelial cells. *Molecular microbiology* **8**, 719-725 (1993).

5 Lappann, M., Haagensen, J. A., Claus, H., Vogel, U. & Molin, S. Meningococcal biofilm formation: structure, development and phenotypes in a standardized continuous flow system. *Molecular microbiology* **62**, 1292-1309, doi:10.1111/j.1365-2958.2006.05448.x (2006).

6 Lappann, M. *et al.* Impact of Moderate Temperature Changes on *Neisseria meningitidis* Adhesion Phenotypes and Proteome. *Infection and immunity* **84**, 3484-3495, doi:10.1128/IAI.00584-16 (2016).

7 Bennett, J. S. *et al.* Independent evolution of the core and accessory gene sets in the genus Neisseria: insights gained from the genome of *Neisseria lactamica* isolate 020-06. *BMC genomics* **11**, 652, doi:10.1186/1471-2164-11-652 (2010).

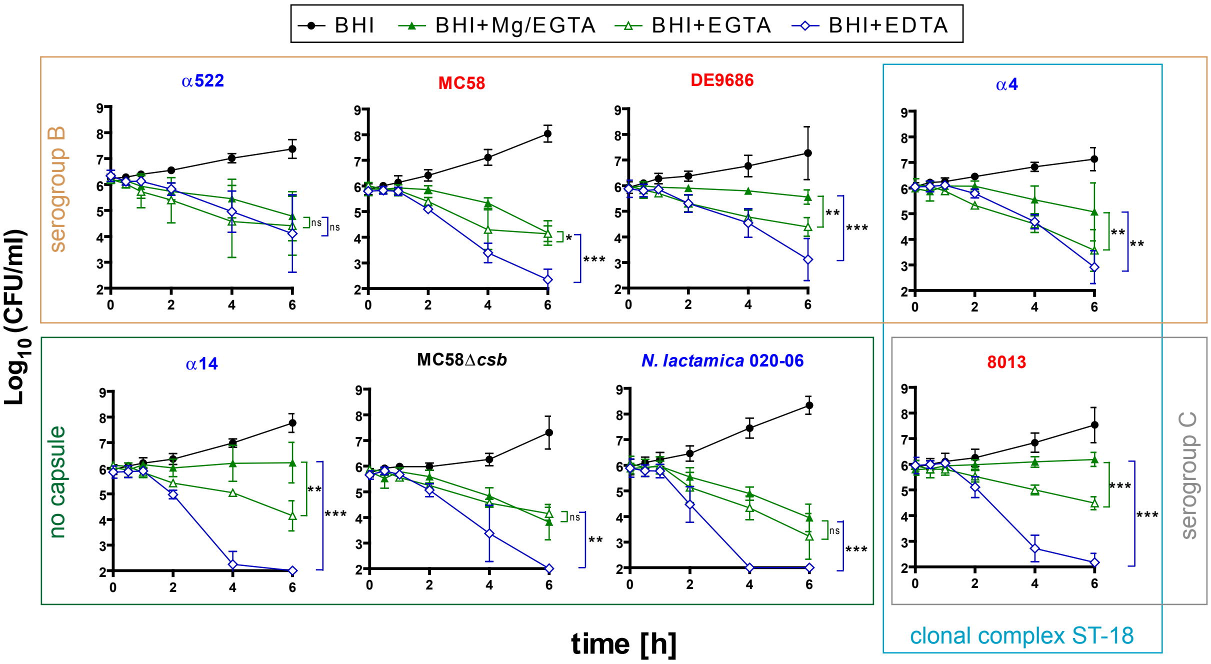

Supplementary figure S1: Effect of Ca^{2+} chelators on bacterial survival in brain heart infusion (BHI) broth. BHI was equipped with either 5 mM EDTA, or 10 mM EGTA or 10 mM EGTA plus 10 mM $MgCl_2$ ('Mg/EGTA') or without calcium chelator (as in legend), then bacteria were added at 10^6 CFU/ml and incubated at 37°C rotating over top. *Neisseria* strains are indicated above graphs; red indicates isolates from invasive disease cases, blue indicates carriage isolates. The graphs are arranged according to the capsule phenotype, serogroup and/or genetic similarity of strains (clonal complexes), as indicated by the boxes. Samples were taken at indicated time points to assess viable counts after plating serial dilutions onto blood agar plates. Plotted are means ± SEM of four independent experiments. *,**,*** indicate $P < 0.05$, 0.01, 0.005 in one-way ANOVA applying Dunnett's multiple comparison *post hoc* test comparing the area under the curve among the conditions with calcium-chelators as indicated by brackets. ns, not significant.

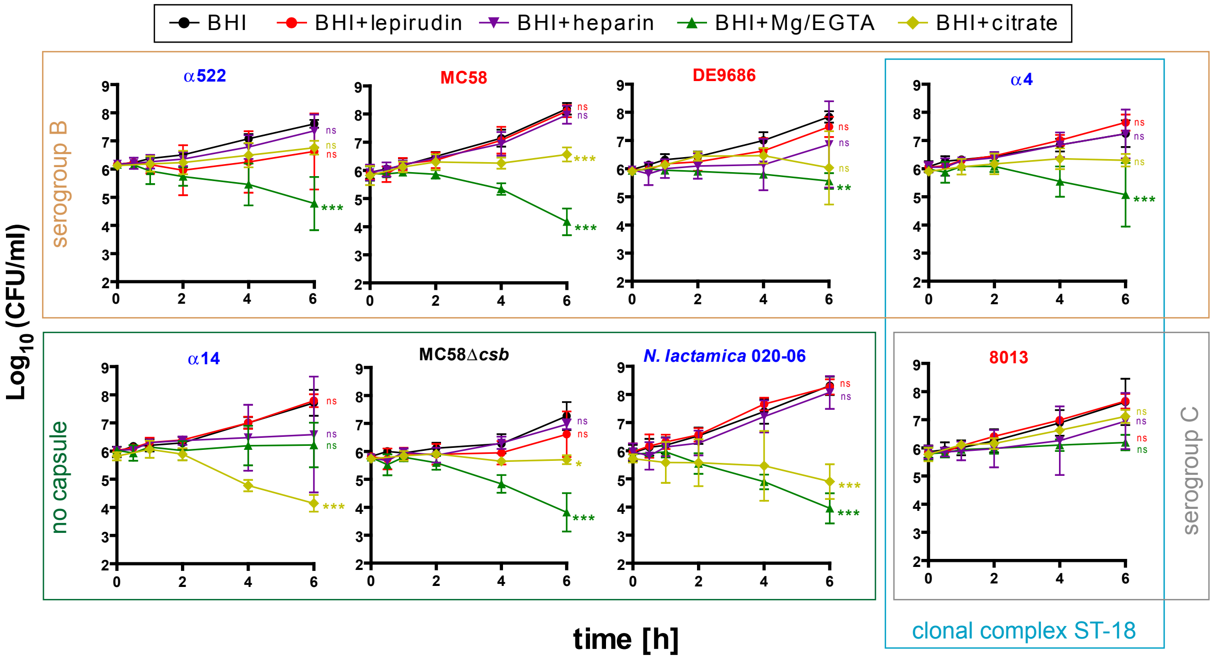

Supplementary figure S2: Effect of anticoagulants on bacterial survival in brain heart infusion (BHI) broth. BHI was equipped with either 10 mM EGTA plus 10 mM $MgCl_2$ ('Mg/EGTA', same dataset as in supplementary figure S1), or with 20 U/ml heparin, or with 3.8 % (w/v) Na_3citrate ('citrate') or with hirudin (525 antithrombin units (ATU)/ml), then bacteria were added at 10^6 CFU/ml and incubated at 37°C rotating over top. Samples were taken at indicated time points to assess viable counts after plating serial dilutions onto blood agar plates. Plotted are means ± SEM of 3-6 independent experiments. *,**,*** indicate $P < 0.05, 0.01, 0.005$ in one-way ANOVA applying Dunnett's multipe comparison *post hoc* test comparing the area under the curve or the differently equipped BHI samples against that of BHI without additives. ns, not significant.

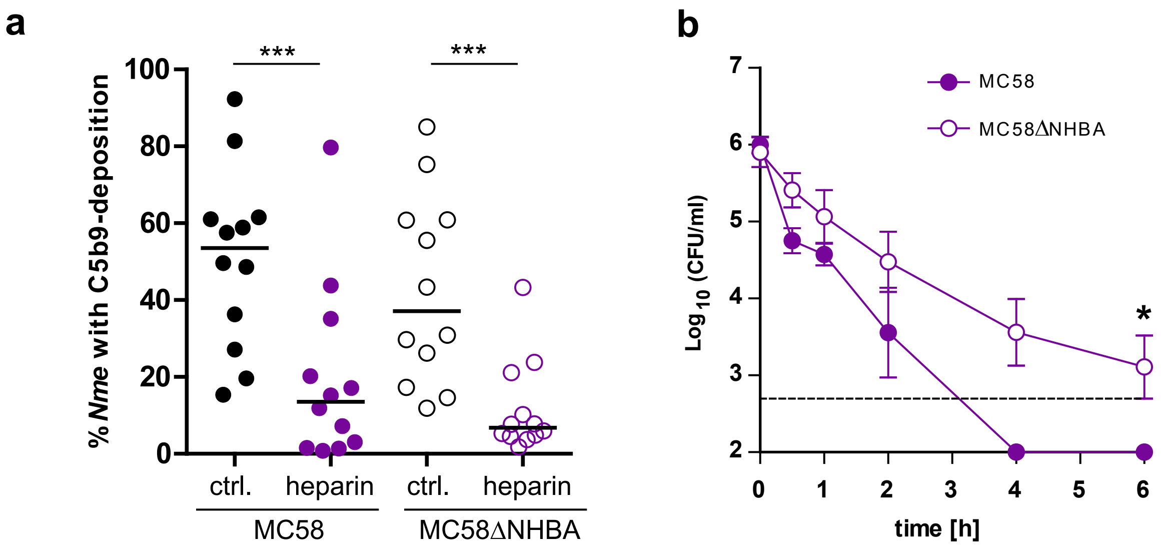

Supplementary figure S3: Role of NHBA in C5b9-deposition and survival in immune serum. (A) C5b9 deposition on strain MC58 and MC58ΔNHBA after incubation with 5 % of immune sera from n = 12 individuals, either in presence or absence of 20 U/ml heparin. *** denotes $P < 0.005$ in repeated measures one-way ANOVA with Tukey's *post hoc* test. **(B)** Survival curves of MC58 versus MC58ΔNHBA in undiluted serum of three immune donors, in presence of 200 U/ml heparin to allow for prolonged survival of the strains. * denotes $P < 0.05$ in Student's T-test comparing the area under the curve.

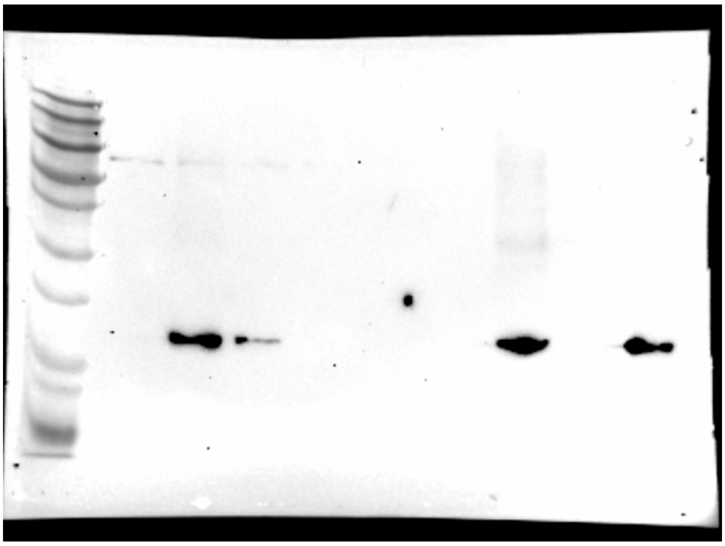

Figure S4: Full image of Western blot to detect Opc in figure 5a. This is a merged image of chemiluminiescence to visualize Opc and regular imaging to visualize the standard (blue protein standard, broad range, New England Biolabs P7706S). Both images were collected on a ChemiDoc MP imaging system (BioRad) in sequence without moving the stained membrane. For chemiluminescence, the ‘chemi/high sensitivity’ mode was used with an exposure time of 221 seconds and signal intensity displayed as negative (i.e. bands appear black, background as white). The regular image of was obtained using the ‘colorimetric’ mode and automatically optimized band exposure time. Contrast and brightness adjustment was kept to a necessary minimum as optimized per default settings of Image Lab software (BioRad); the same program was used for merging of the images.
